# The pathophysiology of prospective memory failure after diffuse axonal injury - Lesion-symptom analysis using diffusion tensor imaging

**DOI:** 10.1186/1471-2202-11-147

**Published:** 2010-11-20

**Authors:** Keita Kondo, Masaharu Maruishi, Hiroki Ueno, Kozue Sawada, Yukari Hashimoto, Tomohiko Ohshita, Tetsuya Takahashi, Toshiho Ohtsuki, Masayasu Matsumoto

**Affiliations:** 1 Hiroshima Higher Brain Function Center, Hiroshima Prefectural Rehabilitation Center, 295-3, Taguchi, Saijo, Higashi-Hiroshima, Japan; 2 Department of Clinical Neuroscience & Therapeutics, Hiroshima University Graduate School of Biomedical Science, 1-2-3, Kasumi, Minami-ku, Hiroshima, Japan; 3 Department of Psychology, Fukuyama University, 958, Sanzo, Higashimura, Fukuyama, Japan; 4 Department of Communication Sciences and Disorders, Hiroshima Prefectural College of Health Sciences, 1-1, Gakuen-chou, Mihara, Hiroshima, Japan

## Abstract

**Background:**

Prospective memory (PM) is one of the most important cognitive domains in everyday life. The neuronal basis of PM has been examined by a large number of neuroimaging and neuropsychological studies, and it has been suggested that several cerebral domains contribute to PM. For these activation studies, a constellation of experimental PM trials was developed and adopted to healthy subjects. In the present study, we used a widely used clinical PM assessment battery to determine the lesions attributable to PM failure, with the hypothesis that lesion-symptom analysis using diffusion tensor imaging (DTI) in subjects with diffuse axonal injury (DAI) can reveal the neuronal basis of PM in everyday life.

**Results:**

Fourteen DAI patients (age: range of 18-36, median 24) participated in this study. PM failure was scored in the range of 0-6 using three sub-tests of the Rivermead Behavioural Memory Test. The PM scores of DAI patients were in the range of 2-6 (median 4.5, inter-quartile range 2.25). The severity of axonal injury following DAI was examined using fractional anisotropy (FA), one of the DTI parameters, at voxel level in each subject. We then obtained clusters correlated with PM failure by conducting voxel-based regression analysis between FA values and PM scores. Three clusters exhibited significant positive correlation with PM score, the left parahippocampal gyrus, left inferior parietal lobe, and left anterior cingulate.

**Conclusions:**

This is the first lesion-symptom study to reveal the neuronal basis of PM using DTI on subjects with DAI. Our findings suggest that the neuronal basis of PM is in the left parahippocampal gyrus, left inferior parietal lobe, and/or left anterior cingulate. These findings are similar to those of previous activation studies with loading experimental PM tasks.

## Background

Prospective memory (PM) is one of the most important cognitive domains in daily life. PM involves remembering to carry out intended actions at appropriate points in the future [1]. Even minimal reflection prompts the realization that the texture of our daily existence is inextricably bound to PM tasks. These tasks include mundane demands such as remembering to pick up bread on one's way home, remembering to mail the letter in one's briefcase, remembering to give one's housemate the message that a friend called, and remembering to load one's bicycle into the car for a ride after work. Theoretically, PM is considered a complex process involving at least four phases. The first phase is planning and encoding intention. The second phase is keeping the intention in mind and monitoring the environment to detect prospective cues while working on other tasks. The third phase is recognition of such cues, while recalling the intention and the intended action. Finally, the fourth is interruption of ongoing activity and then execution of the intended action [1-3]. PM thus appears to be a complex cognitive process depending on both the frontal system and hippocampal system [4]. This theory is supported by the findings of several neuropsychological studies [1-4] and a number of neuroimaging and neurophysiological studies performed to clarify the neuronal basis of PM [5-10]. In these activation studies, experimental PM trials were carefully designed to be adequate psychologically and suitable for PM in real-world situations [3].

Diffuse axonal injury (DAI) is one of the mechanisms of traumatic brain injury (TBI). DAI comprises primary microscopic injury of axons caused by acceleration-deceleration injury force, the pattern of which is more accurately described as multifocal, appearing throughout the deep and subcortical white matter and particularly common in midline structures including the splenium of the corpus callosum and brainstem on post-mortem pathological examination [11]. Cognitive sequelae of DAI are frequently observed, as is PM failure, which leads to problems in everyday life [12-17]. The pathophysiology of the cognitive sequelae of DAI is believed to stem from multiple injury of intracerebral connections. Diffusion tensor magnetic resonance imaging (DTI) has recently emerged as a valuable additional technique for evaluation of survivors with DAI. By measuring the degree of water diffusion anisotropy, quantitative information on the integrity of axons can be obtained at voxel level in the whole brain. It is reported that fractional anisotropy (FA), which is the best rotationally invariant scalar metric for measuring diffusion anisotropy, is broadly reduced in the white matter after DAI [18-20]. Furthermore, DTI parameters are now established as potential quantitative biomarkers for evaluation of the severity of axonal injury and prediction of functional abilities after DAI [19-24].

The purpose of this study was to clarify the neuronal basis of PM. We hypothesised that the pathological lesions contributing to PM failure could be mapped in patients with DAI by performing voxel-based regression analysis between FA values and PM scores. In this study, PM failure was measured by a clinical PM assessment battery, enabling us to determine the neuronal basis of PM in everyday life.

## Results

### Neuropsychological assessments

Basic patient characteristics and cognitive measurements are shown in Table 1. The average verbal intelligence quotient (VIQ) was 80.8 (standard deviation (SD) 18.2), performance intelligence quotient (PIQ) was 79.1 (SD 20.2), and full-scale intelligence quotient (FSIQ) was 78.5 (SD 20.3). The RBMT standardized profile scores were in the range of 7-24 (median 18, inter-quartile range 13.5). The PM scores were in the range of 2-6 (median 4.5, inter-quartile range 2.25). No correlation was observed between PM score and duration from onset (*p *= 0.39) or Glasgow Coma Scale (GCS) at the time of injury (*p *= 0.09). Although a correlation was observed between PM score and years of education (*p *= 0.037), there was no correlation between PM score and VIQ (*p *= 0.19), PIQ (*p *= 0.081), or FSIQ (*p *= 0.12).

**Table 1:** Clinical characteristics and results of cognitive measures in patients with DAI

Age	Gender	Education (years)	Duration (months)	GCS	WAIS-III			RBMT	
						
					VIQ	PIQ	FSIQ	SPS	PM
24	M	16	49	4	75	78	74	14	5
36	M	12	9	3	60	60	57	17	4
27	M	16	15	7	85	74	78	16	4
21	M	12	51	7	77	86	79	22	6
20	M	11	13	3	84	94	87	7	4
19	M	9	6	3	91	97	93	20	4
22	F	12	27	3	n.a.	n.a.	n.a.	8	2
22	M	16	24	7	115	117	118	23	6
34	M	12	3	3	65	60	60	8	3
27	M	16	5	3	111	106	109	22	6
24	M	16	5	3	94	75	88	19	5
18	M	12	3	3	60	47	50	9	2
33	M	14	6	6	63	63	60	23	6
36	M	16	12	3	70	71	68	24	6

^b^24		^a^13.6 (2.4)	^a^16.3 (16.1)	^b^3 (2)	^a^80.8 (18.2)	^a^79.1 (20.2)	^a^78.5 (20.3)	^b^18 (13.5)	^b^4.5 (2.25)

F: female, M: male, GCS: Glasgow Coma Scale, WAIS-III: Wechsler Adult Intelligence Scale-III, VIQ: verbal intelligence quotient, PIQ: performance intelligence quotient, FSIQ: full-scale intelligence quotient, RBMT: Rivermead Behavioural Memory Test, SPS: standardized profile score, PM: prospective memory, n.a.: not applicable. ^a^: mean (standard deviation), ^b^: median (inter-quartile range)

### Voxel-based regression analysis with PM score

**Figure 1:**
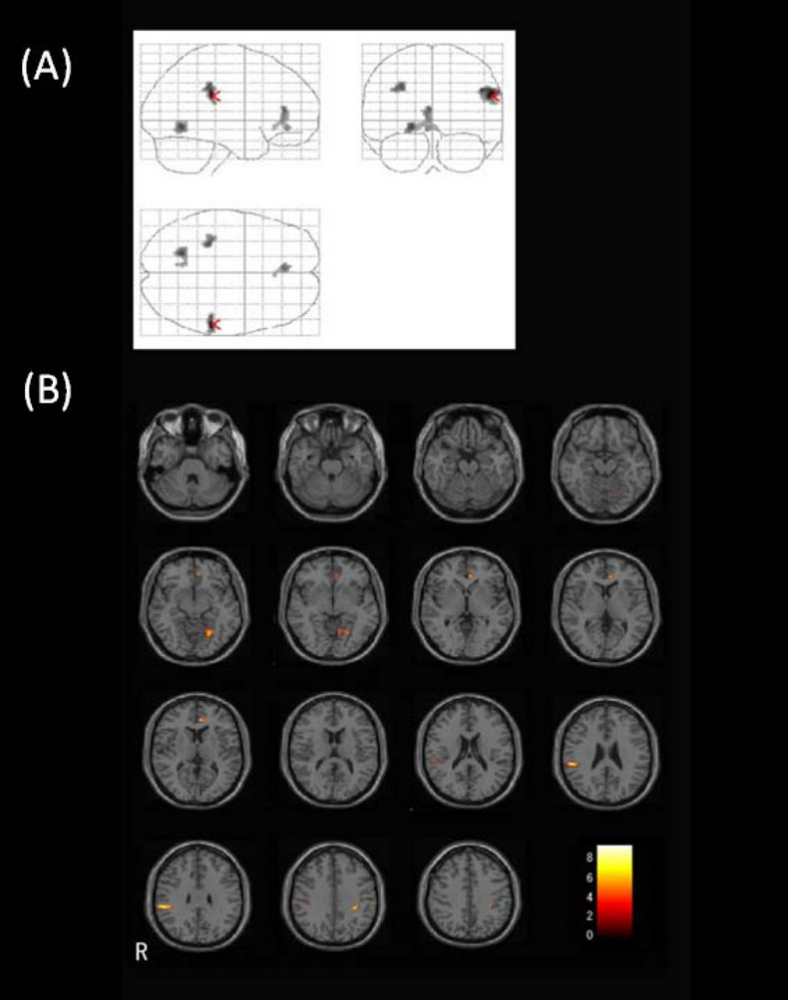
**Results of voxel-based regression analysis between FA value and PM score**. Regression analysis of FA value with a measure of prospective memory in DAI patients revealed four clusters, which are shown on (A) orthogonal projections (red arrowhead indicates the region of global maxima) and (B) axial view of MNI T1 template images. The clusters were observed in the right parietal lobes (Brodmann's area (BA) 40), left parahippocampal gyrus (BA19), left inferior parietal lobe (BA40), and left anterior cingulate gyrus (BA 32). The cluster located in the right inferior parietal lobe was excluded due to its location over the Sylvian fissure. Color bar indicates *T *value. (R = right hemisphere)

Clusters with significant positive correlation with PM score were mapped in the right inferior parietal lobes (peak *T *value = 9.26, peak *Z *score = 4.93, peak Montreal Neurological Institute (MNI) coordinates *x *= 56, *y *= -32, *z *= 26; nearest Brodmann's area (BA) 40), left parahippocampal gyrus (peak *T *value = 6.96, peak *Z *score = 4.33, peak MNI coordinates *x *= -20, *y *= -60, *z *= -8, nearest BA 19), left inferior parietal lobe (peak *T *value = 6.67, peak *Z *score = 4.23, peak MNI coordinates *x *= -30, *y *= -34, *z *= 38, nearest BA 40), and left anterior cingulate gyrus (peak *T *value = 6.34, peak *Z *score = 4.12, peak MNI coordinates *x *= -4, *y *= 38, *z *= 12, nearest BA 32) (Figure 1 and Table 2).

**Table 2:** Covariate regions between FA value and PM score

Cluster size	Peak *T *value	Peak Z score	Peak coordinates			Structure name	Nearest BA
					
			*X*	*Y*	*Z*		
128	7.0	4.3	-20	-60	-8	left parahippocampal gyrus	19
77	6.7	4.2	-30	-34	38	left inferior parietal lobe	40
108	6.3	4.1	-4	38	12	left anterior cingulate	32

*p *< 0.001(uncorrected), minimum cluster size = 50 voxels, voxel size 2 × 2 × 2 mm; *x*, *y*, and *z *values localize regions according to Montreal Neurological Institute stereotactic coordinates. (BA: Brodmann area)

Visual checking of extracted clusters revealed that the cluster located in the right inferior parietal lobe was in the Sylvian fissure, which resulted in lower FA values in subjects whose PM scores were lower. It appeared that reduction of FA in this region did not reflect axonal injury, and instead cortical atrophy. We therefore excluded the cluster located in the right inferior parietal lobe from the group of significant clusters.

**Figure 2:**
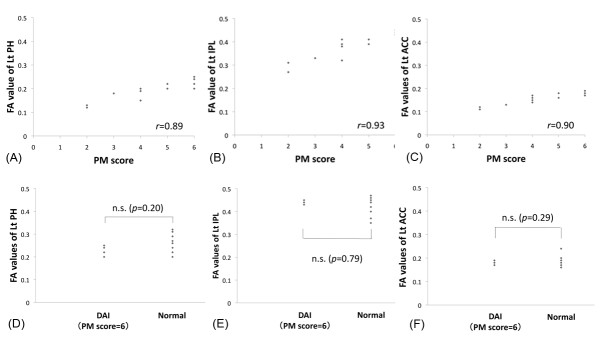
**Correlations between FA value and PM score in each cluster and comparison with normal subjects**. Significant correlations between FA value and PM score were found in (A) the left parahippocampal gyrus (*r *= 0.89), (B) left inferior parietal lobe (*r *= 0.93), and (C) left anterior cingulate (*r *= 0.90). FA values of DAI subjects with full PM score (score = 6) were preserved compared with those of normal volunteers in each cluster (D: left parahippocampal gyrus, E: left inferior parietal lobe, F: left anterior cingulate cortex). (Rt: right, Lt: left, FA: fractional anisotropy, PM: prospective memory, PH: parahippocampal gyrus, IPL: inferior parietal lobe, ACC: anterior cingulate cortex, n.s.: not significant).

The other three clusters located in the left parahippocampal gyrus, left anterior cingulate, and left inferior parietal lobe consisted mainly of subcortical white matter with partial involvement of cerebral cortex. The correlation coefficients between FA value and PM score in each of these clusters was following; left parahippocampal gyrus (*r *= 0.89, *p *< 0.001), left inferior parietal lobe (*r *= 0.93, *p *< 0.001), and left anterior cingulate (*r *= 0.90, *p *< 0.001), respectively. Moreover, there was no significant difference in FA value between DAI subjects with full PM score (score = 6) and normal volunteers in these clusters [left parahippocampal gyrus (*p *= 0.20), left inferior parietal lobe (*p *= 0.79), and left anterior cingulate (*p *= 0.29)] (Figure 2). There were no significant correlation between FA values and times from the injury in each cluster [left parahippocampal gyrus (*r *= 0.33, *p *= 0.26), left inferior parietal lobe (*r *= 0.17, *p *= 0.56), and left anterior cingulate (*r *= 0.18, *p *= 0.54)].

## Discussion

Multiple axonal lesions of varying severity are scattered throughout the white matter in the brains of DAI patients, and various cognitive sequelae are observed depending on the severity and location of such lesions. Since PM function depends on intact intra-cerebral networks in several cognitive domains, disruption of these connections results in the failure of PM. DTI is a suitable method for detecting such disconnections, and is therefore considered useful for identifying certain domains associated with PM. We believe it is possible to determine the brain region attributable to each cognitive function affected in these patients by comparing cognitive parameters and FA values, which can be used to evaluate the severity of axonal injury at voxel level. With this hypothesis, we sought to locate lesions attributable to PM failure after DAI. To the best of our knowledge, no previous study has mapped the lesions attributable to PM failure through voxel-based lesion-symptom analysis using DTI. In this study, three clusters significantly correlated with PM failure were found in the left parahippocampal gyrus, left inferior parietal lobe, and left anterior cingulate.

Of the clusters correlated with PM failure, that in the left parahippocampal gyrus is consistent with the findings of previous neuroimaging studies with loading of experimental PM tasks. In previous PET studies, activation of the left parahippocampal gyrus was observed during loading of experimental PM tasks, and activation of this region was thought to play a role in recognition of cues triggering the performance of intended actions [6,7,10,25]. The second cluster correlated with PM failure was detected in the left inferior parietal lobe. Voxel-based regression analysis with Trail Making Test-B, which was conducted in the same DAI subjects, showed that the cluster correlated with Trail Making Test-B was in the left inferior parietal lobe, in a location close to the cluster correlated with PM (Additional file 1and 2). In functional neuroimaging studies, cortical activation in this area was observed on loading of the Wisconsin Card Sorting Test and Trail Making Test, suggesting that neuronal activity in this region reflects the cognitive process of set shifting [26,27]. Set shifting is believed to participate in PM, e. g. in monitoring of the environment for cues to re-instantiate an intention while performing other tasks (i.e. the second phase of the PM process) or inhibiting other activities at the critical time for performance of the intended action (i.e. the fourth phase of the PM process). It also appeared that lesions in the left anterior cingulate worsen PM function. This finding is consistent with a previous lesion-symptom analysis study using computed tomography. In that study, lesions in the left anterior cingulate causing PM failure were associated with failure of recall of the intention and intended action [2]. These findings suggested that PM failure after DAI might reflect operation of the supporting retrospective components of PM, as well as those related more specifically to maintenance of an intention.

Previous studies using functional neuroimaging and event-related potential recording have revealed a relationship between the right inferior parietal lobe and PM function [1,7-10,28]. In the present study, a correlation between FA value and PM score was found for the cluster in the right inferior parietal lobe. However, post-hoc testing revealed that lower FA values in this cluster could be assigned to the cluster beyond the Sylvian fissure. Further study using accurate voxel-based morphometry is needed to determine whether atrophy of this region contributes to PM failure in patients with DAI.

The localization of the clusters correlated with PM failure was strikingly similar to that of regions activated during experimental PM tasks in previous activation studies, except for anterior prefrontal cortex (aPFC), which has been suggested to be of central importance to PM by several researchers [6-10]. On post-hoc testing, the reduction of FA value in white matter close to aPFC was not significant compared with that in normal controls (data not shown). It is likely that an artifact originating from air in the frontal sinus masked the lesion in aPFC determined by FA value.

The present study is the first to evaluate the neuronal basis of PM using voxel-based regression analysis with FA maps in DAI subjects. An advantage of our methodology is that no experimental PM task was required; we could instead adopt the widely used clinical PM assessment battery. However, among the regions elicited by our method, we could not discriminate that attributable to core PM from that attributable to supportive function for PM.

Other limitations of our study include the following. First, we examined a relatively small number of subjects. Future studies with larger samples of patients are needed to augment the reliability of our findings. Second, DTI data were acquired with diffusion weighting encoded along only 6 independent orientations. To obtain more accurate tensor parameters, a larger number of gradient sampling orientations must be adopted in further studies. Third, the interval between injury and testing varied widely between 3 to 51 months. In a previous study, longitudinal decline of FA values was observed in brains subjected to trauma [29], suggesting that longitudinal changes in FA values may have affected our results. However, post-hoc examination revealed no significant decline in FA value with time from injury. Hence, FA values were not adjusted for time from injury. Despite these limitations, we believe that we were able to map the neuronal basis for PM performance in a daily life situation.

## Conclusions

Using lesion-symptom analysis, we demonstrated that lesions in the left parahippocampal gyrus, left inferior parietal lobe, and/or left anterior cingulate resulted in PM failure. These structures may thus be included in the neuronal circuit of PM. Our findings reinforce those of previous activation studies with loading of experimental PM tasks.

## Methods

### Subjects

Fourteen DAI patients (males 13, female 1, age: range of 18-36, median 24) without focal brain injury participated in this study. They all meet Gennarelli's diagnostic criteria for DAI [30]. Diagnosis was confirmed by the presence of traumatic microbleeding on conventional T2*-weighted MR images [31]. Patients under 18 or over 40 years of age were excluded in order to prevent FA reduction related to lack of completion of development or aging [32]. All participants were right-handed. GCS at the time of injury was 3-7 (median 3, inter-quartile range 2), and all patients were classified as having severe TBI, according to the severity grading advocated by Narayan [33]. All participants with DAI were at least 3 months post-injury, with a mean time from injury of 16.3 (SD 16.1) months. Mean number of years of education was 13.6 (SD 2.4). The Wechsler Adult Intelligence Scale-III test was performed by all DAI subjects. Fourteen healthy age- and sex-matched volunteers without history of TBI, neurological disorder, or psychological disorder were participated. The mean number of years of education was 15.6 (SD 0.4). FA values of healthy participants were measured for the comparison of FA values in the extracted ROI analysis, between those of DAI patients. Informed consent for this study was given by the subject before the experiments. The experimental protocol was approved by the local ethics committee of Hiroshima Prefectural Rehabilitation Center.

### PM assessment

Everyday memory performance was evaluated using standardized profile scores of the Rivermead Behavioural Memory Test (RBMT) in DAI patients [34]. PM failure was assessed using the following three sub-tests of RBMT. First, the belonging sub-test required the participants to ask for a personal belonging, which was taken from them at the beginning of the assessment, when the examiner indicated that the assessment had finished. Participants were also required to remember where the item was concealed. The second measure of PM was the appointment sub-test, in which participants were instructed to ask about a future appointment when a timer sounded. The final measure required the participants to deliver a message at a designated place while completing the RBMT route test. This sub-test had immediate and delayed (30 minutes later) components. In this study, we summed the standardized profile scores of these three sub-tests to obtain a measure of PM failure (scores range 0-6).

### MR data acquisition

All MRIs were performed on a 1.5 T MR scanner (Siemens, 1.5 T Symphony, Erlangen, Germany) in all DAI and normal volunteers. Conventional axial T2-weighted and coronal fluid attenuated inversion recovery (FLAIR) images were obtained beforehand to rule out cerebral contusion or other lesions. Moreover, to confirm traumatic microbleeding, T2*-weighted transverse echo-planar images were obtained (data matrix, 256 × 256 with field of view (FOV) 230 × 230 mm^2^; 20 axial slices; repetition time (TR) 800 milliseconds; echo time (TE) 26 milliseconds; flip angle 20 degrees). DTI was performed using a single-shot spin echo planar imaging technique. Diffusion weighting was encoded along 6 independent orientations and the *b*-value was 1,000 s/mm^2^, together with acquisition without diffusion weighting (*b *= 0). The diffusion-weighted imaging parameters were as follows: data matrix 256 × 256 with FOV 220 × 220 mm^2^; 19 axial slices; 6 mm slice thickness; TR 4600 milliseconds; TE 137 milliseconds.

### Data processing

In this study, DTI data processing was performed using DTI Studio software (Jiang H and Mori S, Johns Hopkins University, Kennedy Krieger Institute) to create FA maps [35]. For voxel-based statistical analysis, first the *b *= 0 images of all subjects were normalized to the standardized MNI T2 template of Statistical Parametric Mapping 2 (SPM2) software (Welcome Department of Imaging Neuroscience, London, UK) using an affine and nonlinear spatial normalization algorithm. Then the FA maps were normalized by applying the normalization parameters determined by normalization of the *b *= 0 images. The spatially normalized FA maps were then smoothed with a 6-mm isotropic Gaussian kernel to improve the signal-to-noise ratio, increase the validity of statistical inference, and improve normalization.

### Statistics

#### Correlations between PM scores and clinical characteristics

Spearman's *r *correlation coefficients were calculated between PM scores and clinical parameters such as years of education, duration from onset, GCS at time of injury, and other neuropsychological scores.

#### Voxel-based regression analysis between PM score and FA value

Voxel-based regression analysis between FA value and PM score was performed using SPM2. In this study, statistical significance was defined by a voxel level threshold set at *p *< 0.001 (uncorrected for multiple comparisons) and clusters with a size larger than 50 contiguous voxels [36-39].

#### Cluster extracted statistics

Each cluster was automatically extracted using software (MarsBaR: http://marsbar.sourceforge.net/). Then, the location of each cluster was visually checked, and the adequacy of its anatomical location confirmed. Spearman's correlation coefficients were determined between the FA value of each cluster and PM score, and the times from injury, respectively. To evaluate whether the FA values were reserved in the patients whose PM performance were normal, we compared FA values in each cluster between DAI patients with full PM score and normal volunteers using the *t*-test.

These calculations were performed with SPSS Statistics for Windows version 17.0. P values less than 0.05 were considered significant unless otherwise indicated.

## Competing interests

The authors declare that they have no competing interests.

## Authors' contributions

KK and MMar contributed to the design of this research. KK and HU performed the DTI data processing and statistical analysis. Neuropsychological assessment was performed by KS and HY. KK, TT, MMar, TOhs, and TOht participated in drafting of this manuscript. All authors read and approved the final manuscript and MMat has given final approval for publication of the final manuscript.

## Supplementary Material

Additional file 1 **Results of voxel-based regression analysis between FA value and Trail making test-B**. This figure demonstrates that the regions correlated with Trail making test-B (TMT-B) scores in the same DAI subjects. Thirteen patients were performed TMT-B, and the mean score was 140.2 (SD 50.6) seconds. There was no correlation between the measures of PM and TMT-B (*r *= -0.371, *p *= 0.213). Regression analysis of FA value with the score of TMT-B in DAI patients revealed three clusters, which are shown on (A) orthogonal projections (red arrowhead indicates the region of global maxima) and (B) coronal view of MNI T1 template images. The clusters were observed in the white matter of left pre-frontal lobes, right anterior cingulate, and left inferior parietal lobe. (C) Compared the cluster correlated with TMT-B (blue) with that correlated with PM (yellow), the cluster located in the left inferior parietal lobe was closed to each other. Color bar indicates *T *value. (R = right hemisphere) Click here for file

Additional file 2 **Covariate regions between FA value and Trail making test-B score**. Regression analysis of FA value with the score of Trail Making Test-B in DAI patients revealed three clusters (x, y, and z values localize regions according to Montreal Neurological Institute stereotactic coordinates, BA = Brodmann area). Click here for file
