# Supplementary figures and images for: The pathophysiology of prospective memory failure after diffuse axonal injury - Lesion-symptom analysis using diffusion tensor imaging
Source: BMC Neurosci. 2010 Nov 20;11:147. doi: 10.1186/1471-2202-11-147 (PMC2998523; doi:10.1186/1471-2202-11-147)

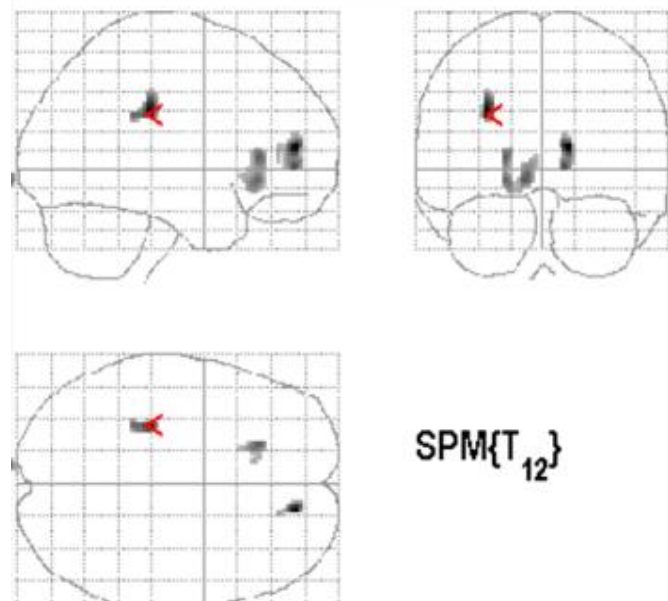

(A)

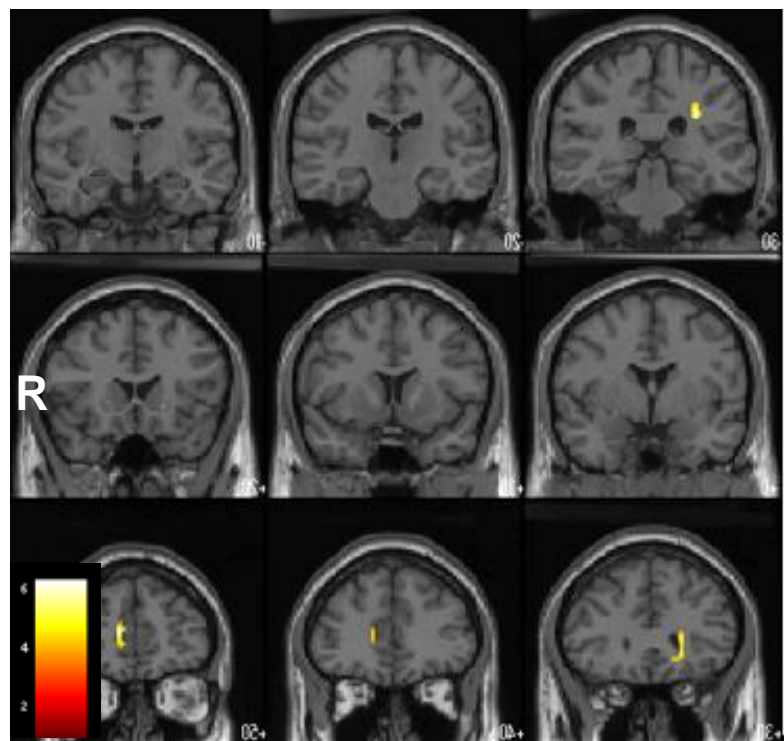

(B)

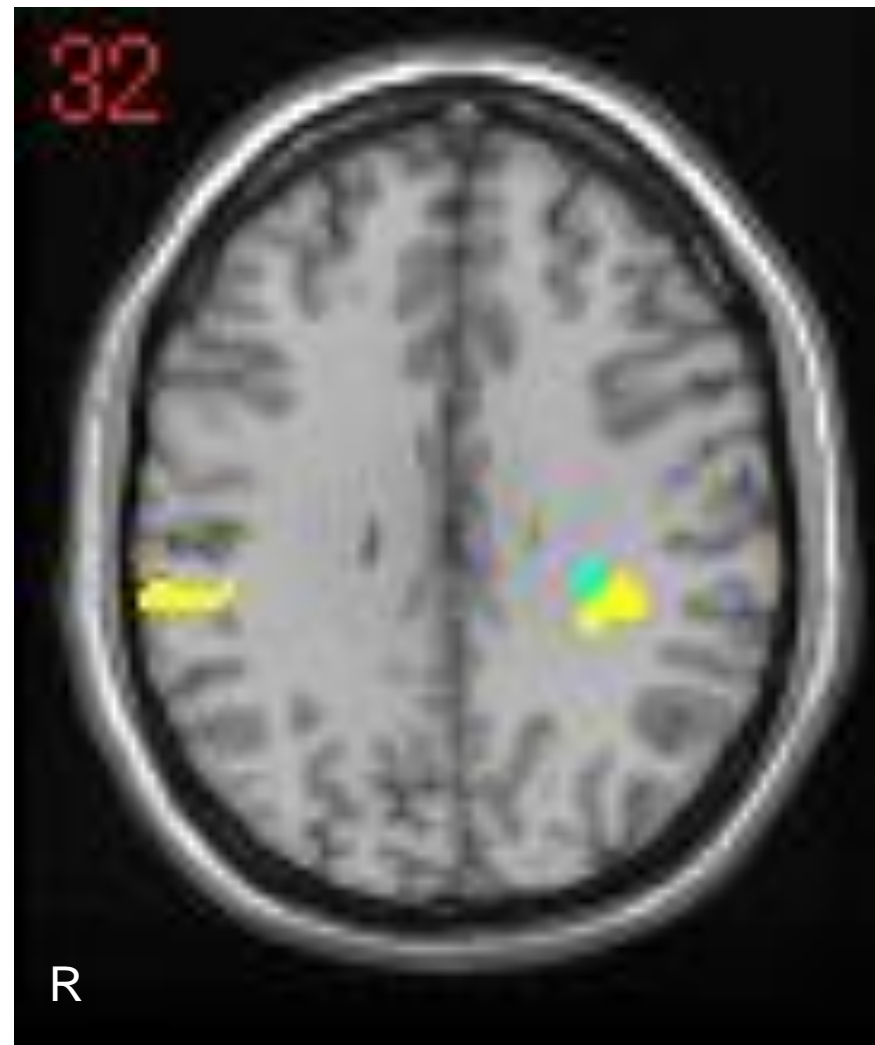

(C)

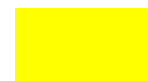

: PM

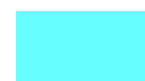

TMT-B

Supplement: Additional file 1 — Results of voxel-based regression analysis between FA value and Trail making test-B. This figure demonstrates that the regions correlated with Trail making test-B (TMT-B) scores in the same DAI subjects. Thirteen patients were performed TMT-B, and the mean score was 140.2 (SD 50.6) seconds. There was no correlation between the measures of PM and TMT-B (r = -0.371, p = 0.213). Regression analysis of FA value with the score of TMT-B in DAI patients revealed three clusters, which are shown on (A) orthogonal projections (red arrowhead indicates the region of global maxima) and (B) coronal view of MNI T1 template images. The clusters were observed in the white matter of left pre-frontal lobes, right anterior cingulate, and left inferior parietal lobe. (C) Compared the cluster correlated with TMT-B (blue) with that correlated with PM (yellow), the cluster located in the left inferior parietal lobe was closed to each other. Color bar indicates T value. (R = right hemisphere) [file 1471-2202-11-147-S1.PDF]
