# Supplementary material for: The pathophysiology of prospective memory failure after diffuse axonal injury - Lesion-symptom analysis using diffusion tensor imaging
Source: BMC Neurosci. 2010 Nov 20;11:147. doi: 10.1186/1471-2202-11-147 (PMC2998523; doi:10.1186/1471-2202-11-147)
Supplement: Additional file 2 — Covariate regions between FA value and Trail making test-B score. Regression analysis of FA value with the score of Trail Making Test-B in DAI patients revealed three clusters (x, y, and z values localize regions according to Montreal Neurological Institute stereotactic coordinates, BA = Brodmann area). [file 1471-2202-11-147-S2.PDF]

cognitive domain	cluster size	peak T value	peak Z score	peak coordinates			structure name	nearest BA
				X	Y	Z		
TMT-B	52	6.31	4.11	-29	-28	27	left inferior parietal gyrus	40
	84	6.29	4.11	14	49	9	right anterior cingulate	32
	81	5.00	3.61	-20	29	4	left cerebrum, sub-nuclear	—
